# Synergistic effects of nanosecond pulsed plasma and electric field on inactivation of pancreatic cancer cells in vitro

**DOI:** 10.1038/s41598-024-51298-y

**Published:** 2024-01-09

**Authors:** Edwin A. Oshin, Zobia Minhas, Ruben M. L. Colunga Biancatelli, John D. Catravas, Richard Heller, Siqi Guo, Chunqi Jiang

**Affiliations:** 1 Frank Reidy Research Center for Bioelectrics, Old Dominion University, Norfolk, VA 23455 USA; 2 Department of Electrical and Computer Engineering, Old Dominion University, Norfolk, VA USA; 3 Department of Medical Engineering, University of South Florida, Tampa, FL 33612 USA; 4 School of Medical Diagnostic and Translational Sciences, College of Health Sciences, Old Dominion University, Norfolk, VA USA

**Keywords:** Cancer, Electrical and electronic engineering, Applied physics, Plasma physics, Biomedical engineering

## Abstract

Nanosecond pulsed atmospheric pressure plasma jets (ns-APPJs) produce reactive plasma species, including charged particles and reactive oxygen and nitrogen species (RONS), which can induce oxidative stress in biological cells. Nanosecond pulsed electric field (nsPEF) has also been found to cause permeabilization of cell membranes and induce apoptosis or cell death. Combining the treatment of ns-APPJ and nsPEF may enhance the effectiveness of cancer cell inactivation with only moderate doses of both treatments. Employing ns-APPJ powered by 9 kV, 200 ns pulses at 2 kHz and 60-nsPEF of 50 kV/cm at 1 Hz, the synergistic effects on pancreatic cancer cells (Pan02) in vitro were evaluated on the metabolic activities of cells and transcellular electrical resistance (TER). It was observed that treatment with ns-APPJ for > 2 min disrupts Pan02 cell stability and resulted in over 30% cell death. Similarly, applying nsPEF alone, > 20 pulses resulted in over 15% cell death. While the inactivation activity from the individual treatment is moderate, combined treatments resulted in 80% cell death, approximately 3-to-fivefold increase compared to the individual treatment. In addition, reactive oxygen species such as OH and O were identified at the plasma-liquid interface. The gas temperature of the plasma and the temperature of the cell solution during treatments were determined to be near room temperature.

## Introduction

Pancreatic cancer is one of the seven leading causes of cancer mortality in the world and is currently one of the most common causes of cancer death in the United States^1–3^. It accounts for about 3% of all new cancer cases in the US and about 8% of all cancer deaths^4,5^. Existing methodologies for cancer treatment have varying limitations and side effects, making it essential to search for new therapeutic techniques. In recent years, cold atmospheric plasmas have emerged as a means to inhibit cancer^6^. They generate reactive oxygen and nitrogen species (RONS) that induce oxidative stress resulting in cell proliferation^7–9^, apoptosis^7,10,11^, and necrosis^12,13^, which suggest their potential in cancer therapy^14–16^. Other biologically active agents that mediate the effect of plasma on cancer cells include electric field, charged particles (ions and electrons), photons, and UV radiation. The degree of their effects on cells is influenced by various parameters such as the plasma working frequency, the voltage waveform, exposure time, and distance from the treated target^17–20^. Among other cold plasmas, nanosecond pulsed plasma jets (ns-APPJ) have the potential to offer great flexibility in treatment dosage with a controlled RONS production modulation^21^.

Besides the atmospheric pressure plasma discharge, nanosecond pulsed electric field (nsPEF) has also been used as a novel method for cancer cell inactivation^22–25^ and tumor ablation^26–28^ by inducing oxidative stress in cells through electroporation^29^. NsPEF treatment has been demonstrated as an effective and safe tumor ablation modality in animal models^24,26,27,30^ and a human trial^31^. It penetrates into the internal structure^32–34^, permeabilizes the membrane, and causes various cellular responses^22,35^. Typically administered at a low pulse repetition frequency, the nsPEF-based ablation technology uses intense nanosecond pulsed electric fields to induce cell death via apoptosis or necrosis^24,26^. Besides membrane permeabilization, other mechanisms such as inhibition of voltage-gated Na^+^ and Ca^2+^ transmembrane current^36^, cell electrodeformation^37^, mechanical stress due to thermoelastic expansion, and ROS formation^38^ were also reported to be associated with the nsPEF-induced bioeffects for electric fields on the order of hundreds of kilovolts per centimeter. However, when the electric field strength becomes too high, unwanted tissue stimulation or damage may occur. Strategies to mitigate the high-field induced side-effect and keep the adjacent normal tissue damage minimal or recoverable are needed. Combining other chemical or physical methods with the nsPEF technology are among the most promising approaches. Wang et al*.* examined the combined effect of nsPEF treatment and a low-concentration chemotherapy drug called gemcitabine on human oral squamous cell carcinoma in vitro^39^*.* They showed that the combination treatment exhibited synergistic actions on necrosis of cancer cells^39^. C. M. Edelblute et al*.* ^40^ used moderate heat to enhance the efficacy of nsPEF for treatment of squamous cell carcinoma, which yielded better tumor regression and a higher survival rate. These combination methods allowed the use of lower electric field strength but achieved higher effectiveness in treatment compared to other treatment groups^29,30^. In addition, other methods including low dose paclitaxel^41^, baicalin^42^, and neutrophil membrane–coated nanoparticles^43^ have also been suggested to combine with nsPEF at mild field strength to increase the treatment efficacy.

It has been recently reported that combining cold plasma with pulsed electric field results in increased treatment efficacy for both plasmid DNA delivery and pancreatic cancer cells (Pan02) inactivation in vitro with relatively mild dosages from both approaches^44^. This study systematically evaluates the synergistic effects of a ns-APPJ and nsPEF on Pan02 in vitro. In addition to quantifying the synergism quotient (SQ) of the combined treatments by assessing the viability of Pan02 cells in suspension after individual and combined ns-APPJ and nsPEF treatments with water-soluble tetrazolium salt (WST-1) assay, electrical cell-substrate impedance sensing (ECIS) was used to monitor the transcellular electrical resistance (TER) of the Pan02 monolayer to determine the cell conditions (e.g., cell migration, permeability, and injury). Although ECIS primarily provides information about cell adhesion, migration, and proliferation, we have employed it in combination with a WST-1 assay to assess the inactivation effects of different physical treatments based on ns-APPJ and nsPEF against Pan02 cells in vitro. ECIS has been used with other assays including cytotoxicity assays and wound healing assays to study cell conditions in real-time^45–48^. The change in resistance indicates the disruption in the confluency of cell monolayers, which is related to the disruption in cell growth possibly due to cell migration, permeabilization or injury. For this study, the TER was monitored over an extended period, e.g., > 40 h, to determine that the disruption or the growth inhibition of the monolayer Pan02 is reversible or irreversible.

To understand the roles played by the reactive plasma species during plasma treatment, optical emission spectroscopy is used to determine the gas phase radicals present in the plasma-liquid interphase. The possible effects of temperature on cell inactivation are assessed with direct measurements of the gas temperature of the plasma, and the solution temperature during treatment. The pH-level is assessed using a pH meter. We demonstrated that treatment with both ns-APPJ and nsPEF induced a synergistic effect on cell inactivation by reducing the metabolic activity.

## Materials and methods

### Experimental setup

**Figure 1:**
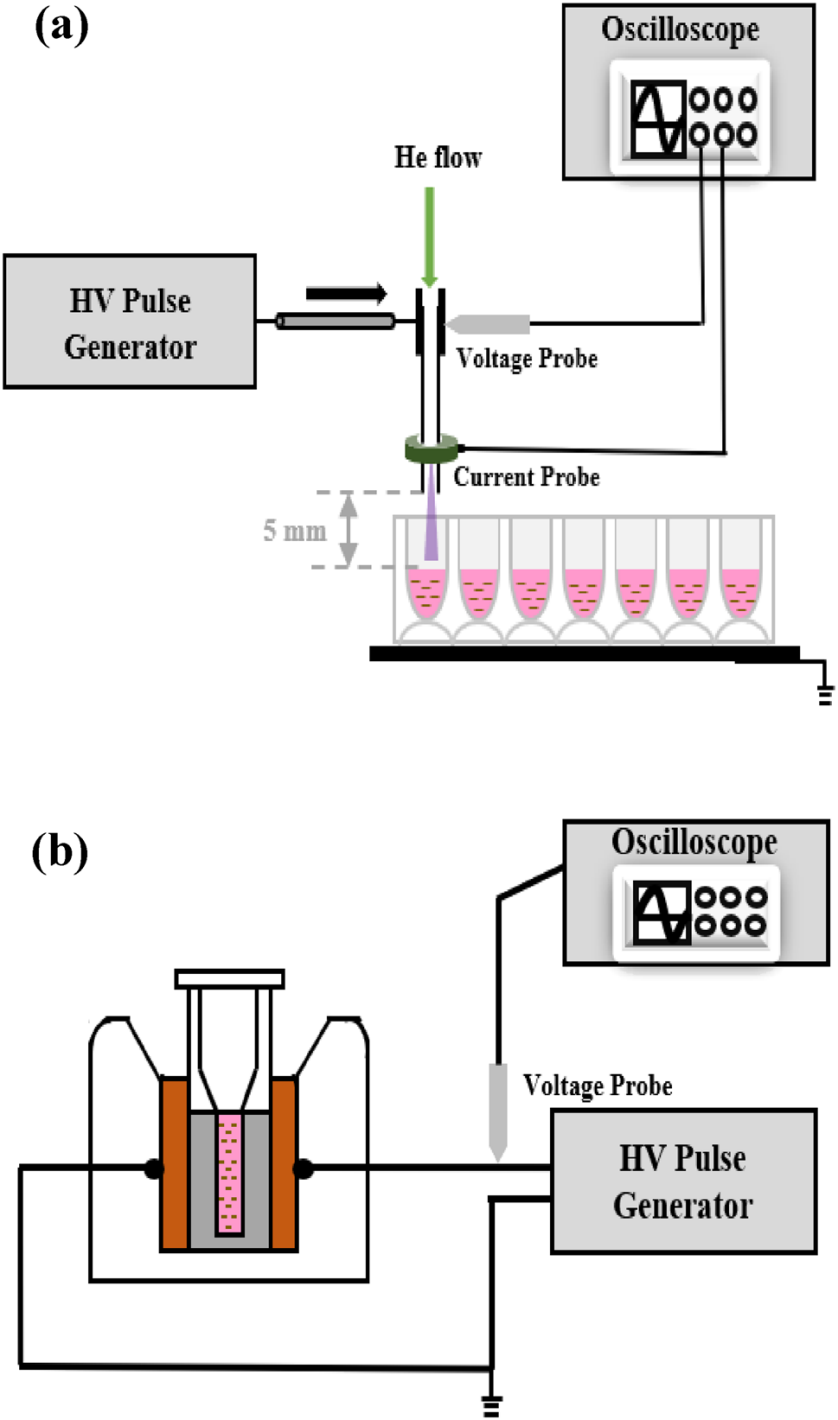
Schematic of the experimental setup of (**a**) 200 ns 9 kV microplasma jet impinging on cell solution in a 24-well plate and (**b**) 60 ns 5 kV nsPEF treating cell suspension in a 0.1 cm cuvette.

The experimental setup of the plasma impinging on Pan02 cell solutions in a 24-well plate is illustrated in Fig. 1a. Details of a similar ns-APPJ system that was used for cancer cell inactivation were previously reported^44^. Briefly, the high voltage electrode is a stainless-steel hollow needle having an inner diameter of 0.26 mm and outer diameter of 0.51 mm. Ultrapure helium (99.9999%, Air Gas), as the working gas, flows through the hollow needle at a flow rate of 355 standard cubic centimeter per minute (SCCM). Although a range of flow rate may be used to generate a steady plasma jet, 355 SCCM was used in this study for maintaining a laminar flow and a stable streamer for the voltage used. The flow rate is controlled by a calibrated rotameter (Matheson FM-1850). The circuit was completed by placing the 24-well plate on a Cu ground plate (Fig. 1a). The plasma is powered by a high voltage pulse generator (DEI, model PVX-4110), which outputs up to 10 kV pulses, less than 60 ns rise and fall times, and up to 10 kHz pulse repetition frequency. A high voltage probe (Tektronix 6015A) was used to measure the load voltage. The discharge current was measured using a current monitor (Pearson 6585) placed around the needle electrode nozzle exit, as shown in Fig. 1a. The gap distance between the needle electrode nozzle exit and the liquid surface was kept constant at 5 mm.

For the nsPEF treatment, a similar setup as described in our previous publications^38,49^ was used. Briefly, a custom-made nanosecond pulse generator was used to generate 60 ns full-width half maximum (FWHM) pulses with repetition frequency up to 10 Hz. The pulse forming line (PFL) of the pulse generator is made of five 50 ohms coaxial cables (Pasternack Enterprises, RG 213/U) connected in parallel to obtain an equivalent matching load of 10 ohms. A spark gap with adjustable gap distance in room air was used as the switch for the PFL. The pulse generator was connected to a parallel plate-based cuvette holder, where a 1 mm-gap cuvette (Biosmith Biotech, Inc, 72,001) may be loaded, as shown in Fig. 1b.

### Cell culture

Pan02 cells were provided by the Division of Cancer Treatment and Diagnosis (DCTD, NCI) and have been maintained in RPMI (1640-1X) (CORNING LOT NO. 16921011) supplemented with 10% fetal bovine serum, antibiotics (100 units/mL penicillin and 100 μg/mL streptomycin, Atlanta Biologicals). Pan02 cells, passage numbers between 4 and 6, were thawed for expansion, and cells with passage numbers between 10 and 20 were used in the in vitro experiments. Pan02 cells were passaged in T75 flasks with 0.5–1 million cells seeded into 16 ml of media and incubated it till 75% confluency was reached. Cell solution at a concentration of 5 × 10^6^ cells/mL was prepared for treatment.

### Ns-APPJ and nsPEF treatment

For all ns-APPJ treatments, the plasma was generated using 200 ns 9 kV pulses at a repetition frequency of 2 kHz. The gap distance between the needle electrode nozzle exit and the liquid surface was kept constant at 5 mm. For the ns-APPJ-only treated groups, while the plasma was powered at the same pulse condition, different treatment times of 0.5, 1, 2, 3, 4, 5, and 6 min were applied. Pan02 cells, 300 µL at a concentration of 5 × 10^6^ cells/mL, were put in a 24-well plate and placed on a grounded plate and exposed to the plasma jet. There was a total of 9 plasma treatment groups that consisted of two negative control and seven plasma-only treated groups. The negative control included one untreated group and one treated with helium flow for 2 min.

For nsPEF treatment, Pan02 cells, 100 uL at a concentration of 5 × 10^6^ cell/mL were placed in a 0.1 cm gap cuvette and pulsed with 60 ns 5 kV (50 kV/cm) pulses at a frequency of 1 Hz. Different pulse dosage was achieved by varying the pulse number between 15 and 90, depending on the experimental design. Because the 0.1 cm gap cuvette only has the capacity to hold 100 µL, three cuvettes were used for each condition to make a total volume of 300 µL used for the plasma-only treatment group.

In the combination treatment, there was a total of nine groups, including five control groups (untreated, helium flow treated, 20 nsPEF pulses only, 2 min plasma only, and 3 min plasma only), and four combined treatment groups (2 min plasma + 20 nsPEF, 3 min plasma + 20 nsPEF, 20 nsPEF + 2 min plasma, 20 nsPEF + 3 min plasma). To assess the treatment order influence on the inactivation of the cells, one group was treated with ns-APPJ first and then with nsPEF, while another group was treated with nsPEF first and then with ns-APPJ. For the combination treatment groups, the time interval between ns-APPJ and nsPEF treatments was monitored and kept constant at 5 min. For all the treatment groups, at least three repeats were conducted for each group.

The parameters used for the treatment protocols of the ns-APPJ generation were chosen to ensure steady and repeatable plasma generation as well as to maintain consistency with our previous work^21^. Also, as studied by^50^, the parameters do not fall in the range of breakdown that could lead to heating-related damage to the cells. Similarly, the parameters for nsPEF treatment were based on the observed linear responses of cells to the number of pulses at the given voltage, pulse repetition frequency, and cells conditions^38^. For the combination treatment, treatment conditions from the ns-APPJ and nsPEF that resulted in mild killing of the cells were selected. This enabled us assess the synergism, and allow safe application of the treatment for future in vivo studies.

### Cell viability assessment with the WST-1 metabolic activity assay

Similarly as described in^35^, the viability of the Pan02 cells was assessed with WST-1 metabolic activity assay. After treatment, the concentration was reduced to 1 × 10^6^ cells/mL. Then 4 µL cell solution was placed into a clear-flat-bottom 96-well plate filled with 96 µL complete medium per well, resulting in 40,000 cells/mL planted in the 0.32 cm^2^ area. Cells were then incubated at 37 °C and 5% CO_2_ for 18 h. After 18-h incubation, 10 µL of WST-1 reagent was added into each well, followed by an additional 2-h incubation. An absorbance Multiskan MCC/340 microplate reader (Fisher Scientific, Hampton, NH) with a test wavelength of 450 nm and a reference wavelength of 630 nm was used to assess the viability of the cells following the formula, treated sample (OD450-OD630)/negative control (OD450-OD630) × 100. Cell solutions without any treatment were used as the negative control. The data was normalized to the control group.

### Measurement of the transcellular electrical resistance of Pan02 monolayers

To assess the stability of Pan02 monolayers, the electric cell-substrate impedance sensing (ECIS®) method was used following treatments. The impedance sensing is achieved by applying a small noninvasive alternating current across the electrodes at the bottom of the ECIS array, which results in a voltage potential across the electrodes. When cells are seeded in the array, they act as insulators, changing the potential across the electrodes, and the impedance of the cells can then be determined^51,52^. To obtain the transcellular electrical resistance (TER) of the monolayer, 300 µL at a concentration of 1 × 10^6^ cells/mL of the treated Pan02 cells were transferred and seeded on electrode arrays (Applied Biophysics, 8W10E +) and placed in an ECIS® model 1600R ζθ (Applied Biophysics). Single frequency/time (SFT) mode was selected at 4000 Hz and at an interval of 600 s. The TER of the monolayer was monitored continuously and normalized for each well’s value at the time of seeding (*t* = 0). Data are presented as mean ± standard deviation (SD) and results were considered significant when *p* < 0.05 with two-way ANOVA and Bonferroni’s Post-test.

### Optical emission spectroscopy of the ns-APPJ impinging on cell solutions

The UV–visible emission of the plasma was collected by a plano-convex lens having a focal length of 75 mm and projected to the entrance slit of a 0.75 m Czerny-Turner monochromator (Princeton instruments, model SP2750) for spectral analysis. A gated-ICCD camera (Princeton instruments, model PI-Max4) which had a 1024 × 1024 pixel array with an individual pixel size of 12.8 µm × 12.8 µm was used as the detector. Spectral dispersion was achieved using a UV–visible holographic grating that has a groove density of 1800 g/mm. The entrance slit width was set to 20 µm. The emission between 300 and 800 nm from the ns-APPJ was collected with a camera exposure time of 8 s, resulting in a total of 8000 accumulations per spectral image. A long pass filter was inserted at the spectrometer slit when taking measurement for wavelengths above 500 nm to remove the spectral interference due to the 2nd orders. A function generator (Standard Research System DG535) was employed to synchronize the ICCD camera and the pulsed power system.

### Statistical analysis

All results were reported as mean ± SD of at least three repeats in each group. Experiments were conducted on different days, and hence the difference among the control groups of different sets of experiments are expected. Statistical analysis was performed for control and treatment groups of each experiment utilizing the One-way ANOVA. A *P*-value of less than 0.05 was considered significant.

## Results

### Power consumption of the plasma source

**Figure 2:**
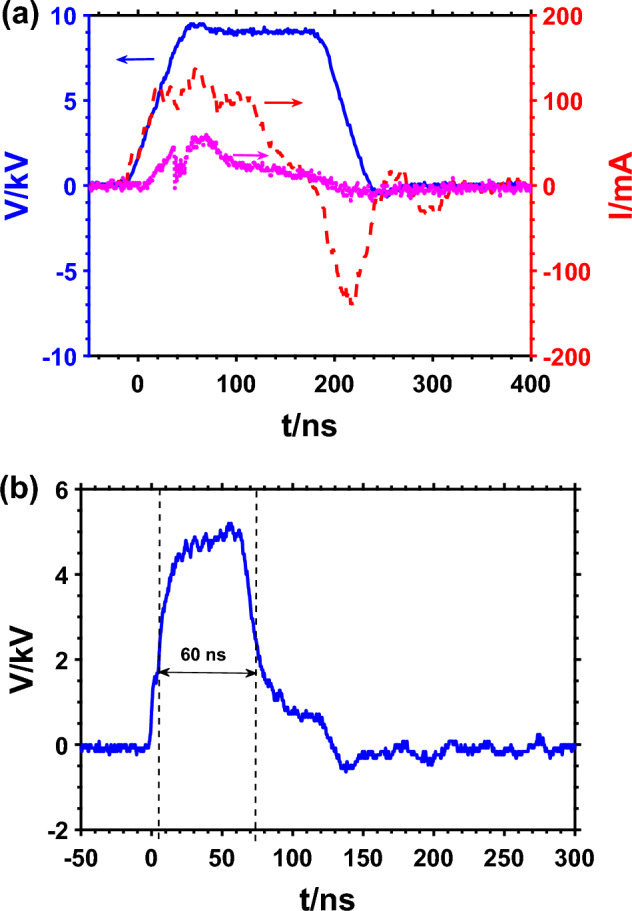
(**a**) Voltage, current and jet current waveforms of microplasma jet impinging on cell solution ( Voltage,  Current,  Jet current) and (**b**) voltage waveform of the nsPEF system during treatment of the cells.

Figure 2a shows the voltage and current waveforms of the plasma jet impinging on cell solution in a 24 well plate. The voltage pulse has a full-width half maximum (FWHM) of 200 ns. The total discharge current was measured using a current monitor (Pearson 6585) placed around the needle electrode nozzle exit. The Jet current which is proportional to the rate of charge transfer from the HV needle electrode to the cells was obtained by subtracting the discharge current measured at the nozzle exit when the helium gas was off from the discharge current when the plasma was on. As previously reported, the onset of the streamer corresponds to the rise of the discharge current^50,53^. As shown in Fig. 2a, the total discharge current was measured to have a peak value of 139 mA and the jet current of about 59 mA. The energy per pulse obtained by integrating the product of the applied voltage and total current over a sufficient period of time was 99 µJ. Also, the delivered charge from the needle electrode to the cell solution is calculated by integrating the jet current over time. The maximum charge delivered was calculated to be 3.6 nC.

Figure 2b shows the voltage pulse generated by the custom-made PFL-pulser. The FWHM of the pulse is 60 ns, and the peak of the voltage is 5 kV, which corresponds to 50 kV/cm of the average electric field between the parallel plates of a 0.1 cm-gap cuvette.

### Inactivation of Pan02 cells by ns-APPJ in vitro

**Figure 3:**
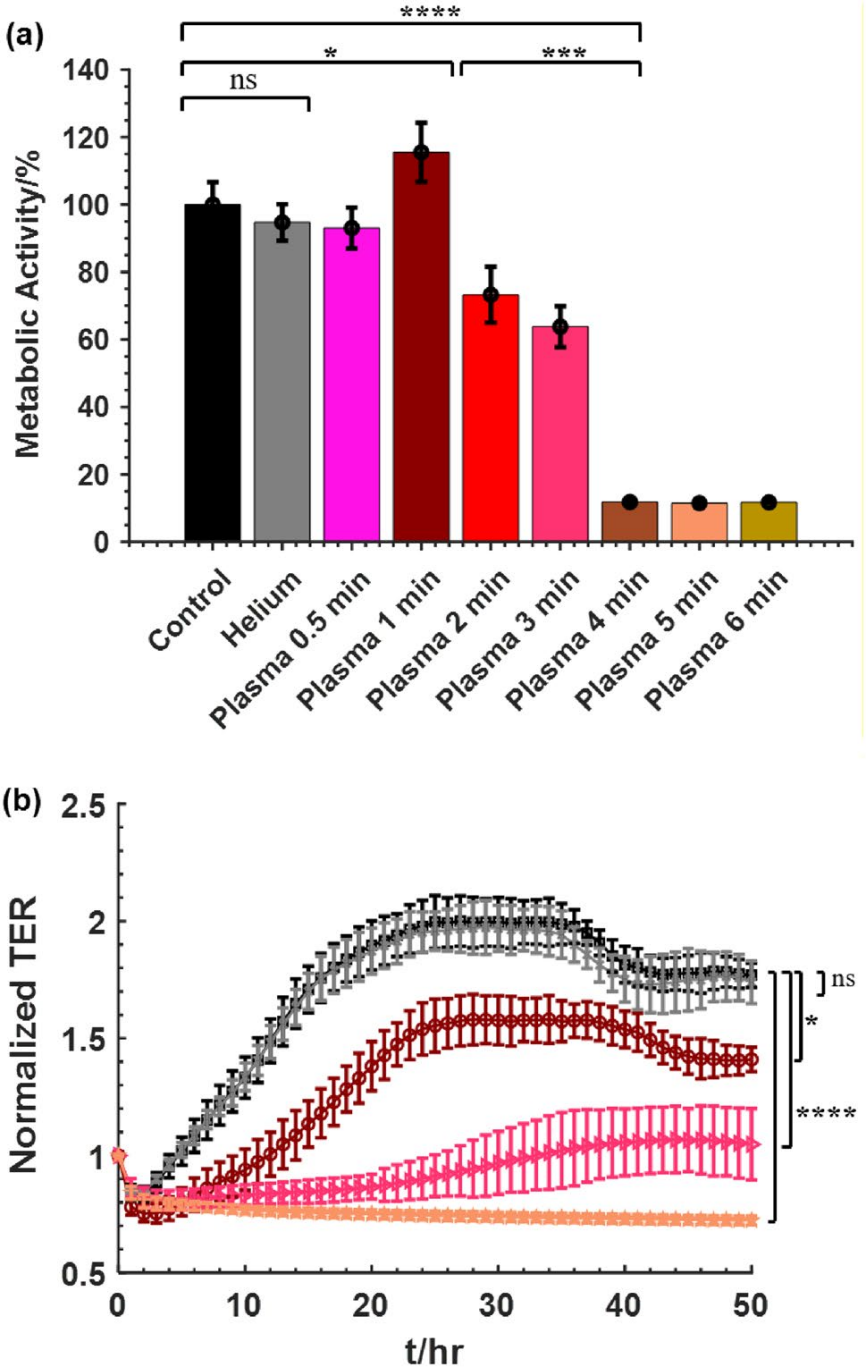
Effect of ns-APPJ on the viability of Pan02 cells. Cells at a concentration of 5 × 10^6^ cells/mL in a 24 well plate were treated with different doses of nsAPPJ; control, helium, plasma 30 s, 1 min, 2 min, 3 min, 4 min, 5 min, 6 min. (**a**) Cell viability measured by WST-1 metabolic activity assay and (**b**) normalized TER measured for 48 h after treatment. ( Control,  Helium,  Plasma 1 min,  Plasma 3 min,  Plasma 5 min). Means ± SD; n = 3–5; *p < 0.05; ***p < 0.001; ****p < 0.0001.

Pan02 cells in suspension in a 24-well plate were exposed to different plasma dosage by varying the treatment time, 0.5, 1, 2, 3, 4, 5, 6 min. A dosage dependent response to the plasma treatment time on metabolic activity was observed, as shown in Fig. 3a. Exposing the cells to helium flow or plasma for 0.5 min had no significant impact on cell activity. Increasing the treatment time to 1-min resulted in 15% increase in the metabolic activity while 2 min and 3 min resulted in 27% and 37% cell death respectively. Longer than 3-min treatment resulted in higher killing rate; 90% inactivation of the cells was obtained after 4 min plasma treatment. Further increasing the treatment time to 5 min or 6 min, however, did not result in further decrease in cell viability, suggesting a saturated inactivation effect by the plasma.

A similar dosage dependence of the ns-APPJ treatment on the TER of the Pan02 cells was also observed, as shown in Fig. 3b. Treatment by helium had negligible effect on the TER of Pan02 cells. Exposing the cells to 1 min of ns-APPJ treatment resulted in a small, non-significant, decrease in TER compared to the control. In both groups, confluency was reached at approximately 24 h. For ns-APPJ treatment longer than 3 min, the TER shows minimal resistance and stunted recovery of resistance, indicating inactivation of majority of cells. The longer the treatment time, the lower the TER of the cells was observed, signifying that more damage was caused to the cells.

### Inactivation of Pan02 cancer cells by nsPEF in vitro

**Figure 4:**
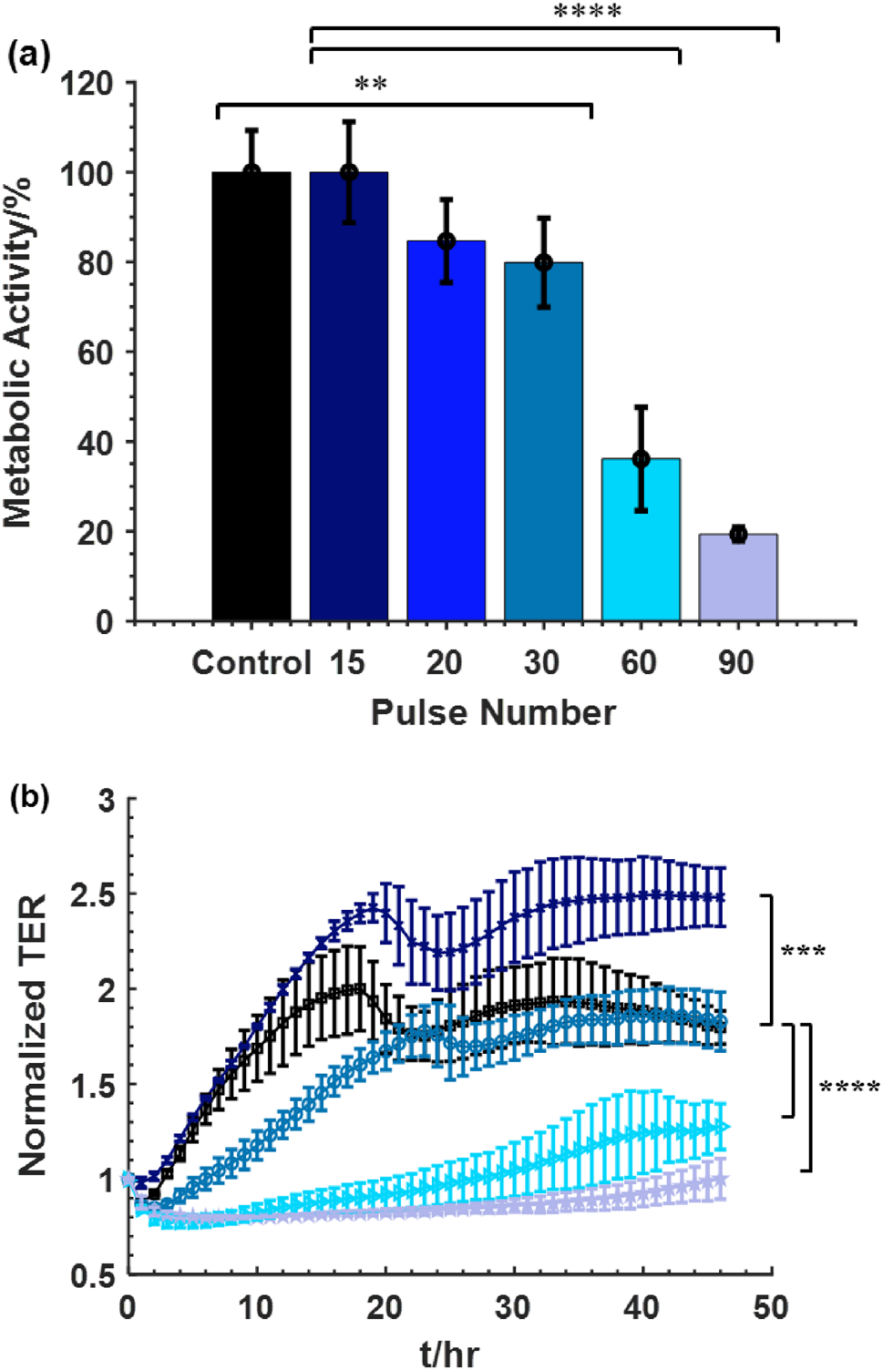
Effect of nsPEF on the inactivation of Pan02 cells in vitro. Cells at a concentration of 5 × 10^6^ cells/mL in a 0.1 cm gap cuvette were treated with different doses of nsPEF: control (0), 15, 20, 30, 60, 90 pulses. (**a**) Cell viability measured by WST-1 metabolic activity assay at 24 h after treatment and (**b**) normalized TER measured for 48 h after treatment. ( Control, 15 pulses,  30 pulses,  60 pulses,  90 pulses). Mean ± SD; n = 3–5; **p < 0.01; ***p < 0.001; ****p < 0.0001.

The viability of the Pan02 cells in suspension with respect to different nsPEF pulse numbers is shown in Fig. 4a. A dosage dependence of the pulse number on the viability, from 15 to 90 pulses, was observed. Exposing the cells to 15 pulses had negligible impact on the metabolic activity comparing to the negative control group without treatment. Applying 20 pulses and 30 pulses resulted in 15% and 20% cell death respectively. Increasing the number of pulses led to more cell death with 64% cell death after 60 pulses treatment and 81% after 90 pulses treatment.

Figure 4b shows the normalized TER of Pan02 cells after nsPEF treatment. When 15 pulses were applied, the TER increased slightly compared to the control. This could suggest that low dose nsPEF increased the monolayer integrity. For the 30 pulses-treated group, the TER between 10 and 20 h after treatment differs significantly from the control group as it shows delayed confluency. The delayed confluency suggests the cells fail to recover quickly after the disruption caused by nsPEF, possibly due to increased permeabilization in the cell membrane. Cells treated with 60 and 90 pulses show significant decrease in the TER and remained low for the duration of the measurement. The higher the pulse number, the lower the TER.

### Inactivation of Pan02 cells by combined treatment of the ns-APPJ and nsPEF in vitro

**Figure 5:**
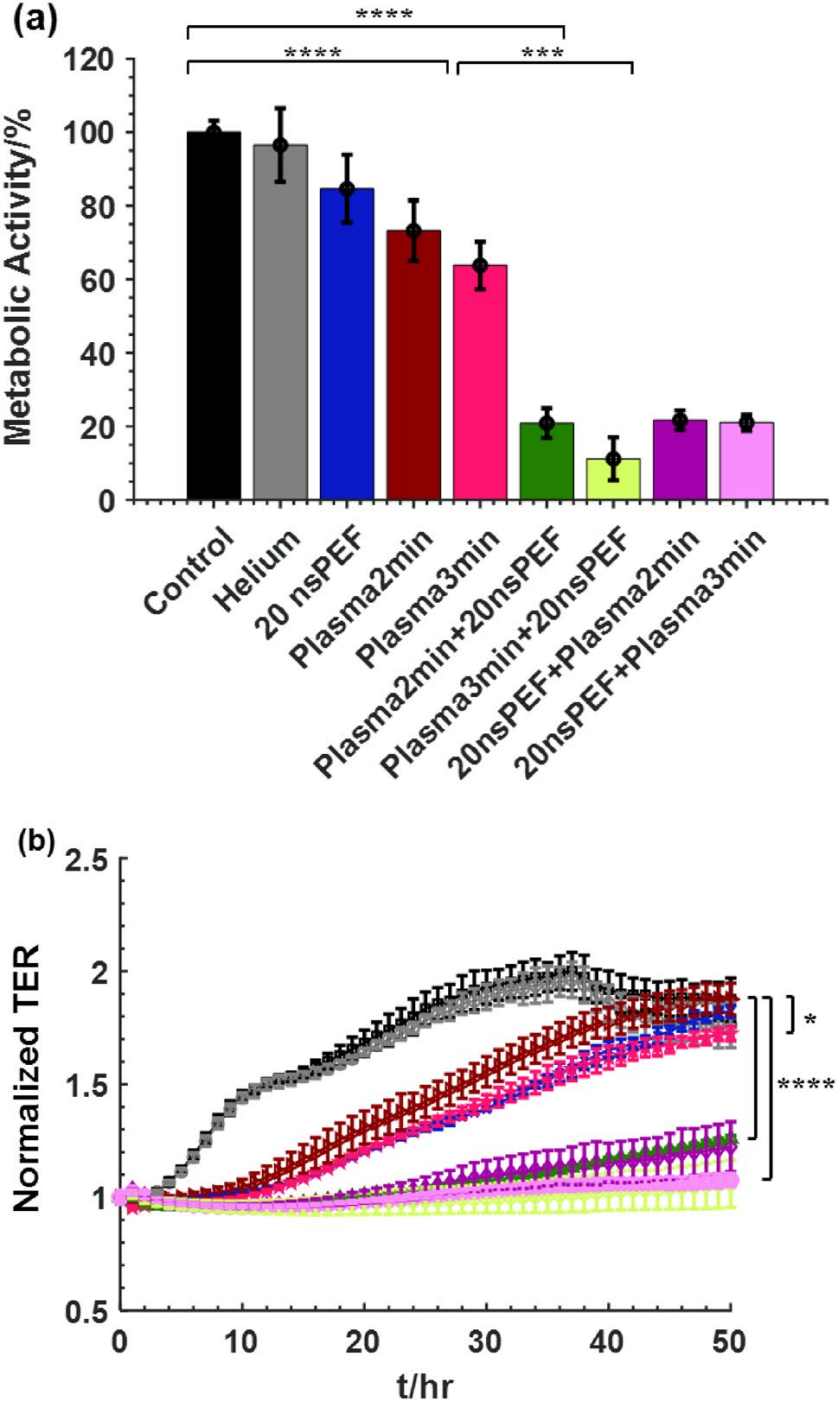
Effect of combinational treatment of plasma and nsPEF on Pan02 cells. Cells were treated with plasma alone or in combination with nsPEF. (**a**) Cell viability by WST-1 metabolic activity assay was determined 24 h after treatment. (**b**) normalized TER obtained for 48 h after treatment. ( Control,  Helium,  20 nsPEF,  Plasma 2 min,  Plasma 3 min,  Plasma 2 min + nsPEF,  Plasma 3 min + nsPEF,  nsPEF + Plasma 2 min,  nsPEF + Plasma 3 min). Mean ± SD; n = 3–5; *p < 0.05; ***p < 0.001; ****p < 0.0001.

The viability of the Pan02 cells in suspension after treatment at different conditions is shown in Fig. 5a. Exposing the cells to helium flow for 2 min did not have any significant effect on the cells. Separately applying 20 pulses, 2 min ns-APPJ and 3 min ns-APPJ resulted in cell death of 15%, 27%, and 37% respectively. Applying the combination treatment of 2 min ns-APPJ + 20 nsPEF, 3 min ns-APPJ + 20 nsPEF, 20 nsPEF + 2 min ns-APPJ, and 20 nsPEF + 3 min ns-APPJ resulted in 79%, 89%, 78%, and 79% cell death respectively. The combination treatment had more significant effect on cell inactivation by increasing cell death by 3–3.5-fold compared to the nsPEF treatment alone. The TER for the single and combination treatment of nsPEF and ns-APPJ is shown in Fig. 5b. Exposing the cells to helium flow for 2 min had no significant impact on the TER. Applying 20 nsPEF to the cells showed delayed confluency, indicating the cell’s inability to recover quickly. This delayed confluency was also observed in the groups with 2 min ns-APPJ treatment and 3 min ns-APPJ treatment, indicating a reduction in the cells ability to recover. Combined nsPEF and ns-APPJ resulted in flat TER values, indicating complete cellular inactivation and lack of monolayer confluency.

### The emission spectrum of the ns-APPJ

**Figure 6:**
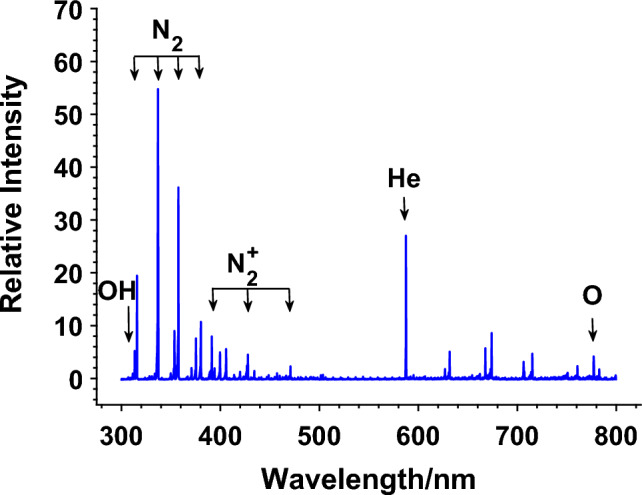
Integrated optical emission spectra of single needle plasma jet impinging on Pan02 cells in Vitro.

The emission spectrum of the ns-APPJ revealed the production of certain excited plasma species in the gas phase, which may have played important roles during the plasma inactivation of the cells. Figure 6 shows a spatially integrated emission spectrum, where the line intensity was integrated along the z-axis from the nozzle tip to the liquid surface. Several electronically excited species were generated in the plasma including OH (A^2^Σ^+^), N_2_ (C^3^Π_u_), N_2_^+^ (B^2^Σ_u_^+^), He (3^3^D), and O (3p^5^P). Among them, reactive oxygen species such as OH and O are known to play important roles in activating Redox chemistry^54^.

### Temperature measurements

The potential heating effect due to the ns-APPJ and the nsPEF treatment was investigated. For the ns-APPJ treatment, this was investigated by measuring the gas temperature just above the cell solution surface and the solution temperature just below the plasma-liquid interface. In addition, the temperature for the nsPEF treatment was assessed by measuring the temperature of the solution. All temperature measurements were obtained during treatment.

**Figure 7:**
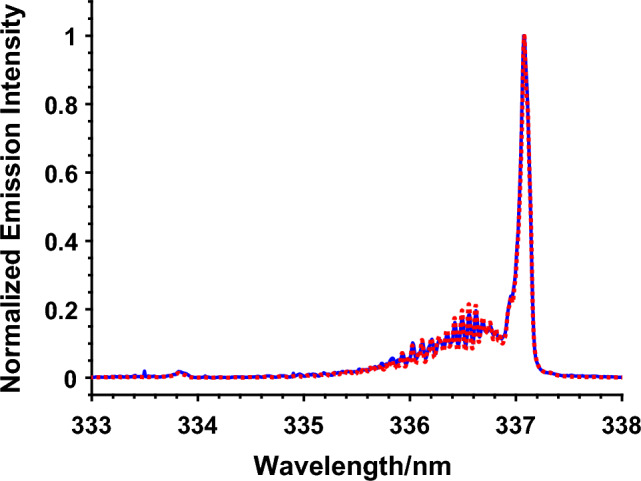
Experimental and fitting emission spectra of N_2_ second positive system to determine the rotational temperature of the ns-APPJ powered by 200 ns 9 kV pulses at 2 kHz, impinging on cell solution. Rotational temperature obtained is 300 K. ( Experimental,  Model).

To obtain the gas temperature, the rotational temperature of the N_2_ second positive system along the axis center of the plasma jet was measured. Since N_2_ is the majority gas molecule in air, the rotational temperature of the excited state N_2_(C) has been commonly used to assume the ground state temperature. The rotational temperature was determined by fitting the emission spectra of the N_2_ (C–B) transition with a simulated emission line using Specair®^55,56^, as shown in Fig. 7. The experimentally measured emission spectrum was obtained from the line-integration of the emission in the axial direction over the entire length of the plasma jet. The temperature of 300 ± 25 K obtained here is hence the average gas temperature of the plasma at the axial center.

Furthermore, a fiber optics temperature probe (Rugged Monitoring H201) was inserted at the bottom of the cell solution to measure the temperature of the solution. The diameter of the probe was 750 µm. Three repeats for each measurement were carried out. Ns-APPJ treatment for the longest treatment time of 6 min used in this study only showed increased temperature of 2 °C from 21 to 23 °C (accuracy of ± 0.8 °C), equivalent to 296 K. This agrees well with the temperature measurement obtained from spectroscopic measurement. Furthermore, during the nsPEF treatment, only a 1.5 °C increase from 21 to 22.5 °C (accuracy of ± 0.8 °C) was recorded after applying 90 pulses, equivalent to 296 K. In both cases, the temperature change was small and not sufficient to cause any heat related damage to the cells.

## Discussion

This work reports the combination effect of a ns-APPJ and nsPEF on pancreatic cancer cells in vitro. To investigate the synergism of two techniques, the experimental conditions are critical. If the dosage of either treatment technique is too high, little information can be extrapolated when the second treatment is added, as most of the cells are already inactivated. Thus, we have employed doses of ns-APPJ and nsPEF that evoke mild effects in cells for the combinational treatment. The combined treatments result in higher Pan02 cell killing than the sum of the individual treatment applied at the same condition. In particular, treatment with 2-min ns-APPJ followed by 20 nsPEF achieved higher killing rate (~ 80%) compared to the sum (~ 42%) of the individual treatments at the same condition. In other words, the synergistic effect resulted in approximately 38% more cell death compared to the cumulative effect of the individual treatments, as shown in Table 1. This synergistic effect was also observed in the TER measurements where the combinational treatment resulted in more reduction in the TER compared to the sum of the individual treatments.

**Table 1:** Percentages of the inactivated cells obtained by cumulative and combinational treatments at different treatment conditions along with the corresponding synergism quotient.

Treatment condition	Cell inactivated (%) (Mean ± SD) for Cumulative treatments, (A + B)	Cell inactivated (%) (Mean ± SD) for combinational treatment, (AB)	SQ
2 min ns-APPJ + 20 nsPEF	42.09 ± 8.7	79.11 ± 4.0	1.75
3 min ns-APPJ + 20 nsPEF	51.53 ± 7.8	88.82 ± 5.8	1.71
20 nsPEF + 2 min ns-APPJ	42.09 ± 5.1	78.29 ± 2.6	1.74
20 nsPEF + 3 min ns-APPJ	51.53 ± 4.7	78.99 ± 2.1	1.50

To quantify the degree of the synergistic effects induced from the different treatment techniques, synergism quotient (SQ), which was previously introduced by others^39^, can be used here. The SQ of combined treatments is obtained by subtracting baseline values from all treatments and then dividing the effects of combined treatments by the sum of individual treatments, as shown in the simplified equation below:

1 $$SQ=\frac{AB}{A+B}$$

where AB is the effect of combined treatment, A is the effect of the first treatment technique, and B is the effect of the second treatment technique.

Table 1 shows the inactivated cells in percentage after cumulative and combinational treatments following the WST-1 assay and the corresponding SQs. The SQs obtained from treatments of 2-min ns-APPJ + 20 nsPEF and 3-min ns-APPJ + 20 nsPEF are approximately 1.75 and 1.71, respectively. When the treatment order is switched, the SQs for 20 nsPEF + 2-min ns-APPJ and 20 nsPEF + 3-min ns-APPJ are approximately 1.74 and 1.50, respectively. Considering the error of the measurements, < 20%, the difference among the combination groups is not significant.

In addition, the SQ value due to ns-APPJ and nsPEF is relatively high comparing to many of the reported work from other techniques. Wang et al^39^. combined nsPEF and low dose of gemcitabine for treating human squamous cell carcinoma in vitro and observed 1.03 unit of SQ for treatment parameters similar to that used in this study. Also, Pefani-Antimisiari et al^57^. combined ns-APPJ and liposomal doxorubicin for treating melanoma cells in vitro and obtained SQ of 1.0.

The presence of synergism suggests that the mechanism of action of each treatment modality are likely through different pathways or at different sites. For low dose ns-APPJ (e.g., 1 min plasma exposure), the cell metabolic activity was increased 15% but TER was reduced. We hypothesize that a relatively low dose treatment causes moderate cell proliferation but also increases permeabilization. Low dose of plasma treatment stimulated cell growth was also reported by others^58^. Higher dose ns-APPJ (e.g., > 2 min plasma exposure), however, causes the death of the cells. The heating in cells and cell medium due to the plasma treatment was not significant because the temperature remained near room temperature and below the hypothermia temperature^59^. Plasma-induced heating was hence not sufficient to induce any damage to the cells. The pH of the cell solution also remained neutral through the treatment. Previous studies have shown that ROS were generated at the plasma-substrate interface and that the amount of ROS produced increased with plasma exposure time^21,60–62^. In this work, RONS such as O, OH, and N_2_ were observed in the plasma and at the plasma-liquid interface, indicating that RONS may play significant role in the cell inactivation via oxidative stress^44^. Note that the OES-based diagnostic technique only identifies the excited species generated above the liquid surface and cannot directly measure the radicals formed in the liquid. However, it is known that these gas-phase RONS species are transferred to the liquid and form radicals in the treated liquid media^21^. Radicals such as hydrogen peroxide (H_2_O_2_), nitrite (NO_2_^−^), nitrate (NO_3_^−^), and peroxynitrite (ONOO) were previously detected in the plasma-activated liquid and considered playing important roles in plasma-induced cellular effects^63–65^.

With a low dose nsPEF (e.g., 15 pulses), cell viability remained the same but the TER increased, suggesting that the low dose nsPEF had no significant effect on cells or might have induced mild proliferation^66^, but were not sufficient to induce regulated cell death^67^. When higher doses of the nsPEF (> 20 pulses) were applied, cell death occurred, and the Pan02 survival rate decreased with pulse number increasing. It is evident that the pulse number or dosage of the nsPEF plays an important role in cancer cell inactivation possibly through reversible or irreversible electropermeabilization of the membrane. Additionally, the temperature of the cell solution during the nsPEF treatment remained at room temperature, suggesting there is no heat related damage to the cells.

The synergistic effects against Pan02 cancer cells in vitro for the combined treatments with both low doses of ns-APPJ and nsPEF are evident, although the treatment order did not seem to introduce significant difference on the inactivation results. We speculate that a low dose of nsPEF induces oxidative stress on cells by permeabilizing the membrane, and applying ns-APPJ treatment of a mild dose allows the plasma-induced exogenous RONS to more readily penetrate into the cell, resulting in direct attack on nuclei or mitochondria^68^ or allowing more exogenous RONS to invade the nuclei^6,38^. The combined treatment with both the plasma-induced exogenous RONS and nsPEF-induced intracellular ROS may result in an efficient pathway to surpass the threshold of oxidative stress of cells hence resulting in a synergistic enhancement in cancer cell inactivation.

Further studies are needed to better understand the synergistic effect of plasma and nsPEF on cells. Quantitative measurements of the exogenous and intracellular RONS, mitochondrial membrane potential, and the associated bioeffects are needed to explore biological safety, as well as to better understand and optimize the treatment technique in both in vitro and in vivo models. Importantly, the specificity of the synergistic effect to cancer cells will need to be examined with different types of cells and tissue including both healthy and cancerous ones. After all, this in vitro study is only a first step towards applying this novel approach to a clinical environment where the type and condition of the tumor such as tissue hardness and conductivity may affect the treatment outcome. More quantitative and mechanism studies using in vivo models are needed to develop this technique for the next-step clinical trials.

## Data Availability

All data generated or analyzed during this study are included in this paper. Any additional data or subset of datasets used for this study are available from the corresponding author upon request.
